# Glycoprotein Matrix Zinc Exhibits Improved Absorption: A Randomized Crossover Trial

**DOI:** 10.3390/nu16071012

**Published:** 2024-03-30

**Authors:** Ralf Jäger, Martin Purpura, Jaci Davis, Nikolas Keratsopoulos, Mandy E. Parra, Ariane H. Secrest, Grant M. Tinsley, Lem Taylor

**Affiliations:** 1 Increnovo LLC, Whitefish Bay, WI 53217, USA; ralf.jaeger@increnovo.com (R.J.); martin.purpura@increnovo.com (M.P.); 2 Human Performance Lab, School of Exercise and Sport Science, University of Mary Hardin-Baylor, Belton, TX 76513, USA; jaci.davis@umhb.edu (J.D.); njkeratsopoulos@mail.umhb.edu (N.K.); mparra@umhb.edu (M.E.P.); 3 School of Health Professions, University of Mary Hardin-Baylor, Belton, TX 76513, USA; asecrest@umhb.edu; 4 Energy Balance & Body Composition Laboratory, Department of Kinesiology & Sport Management, Texas Tech University, Lubbock, TX 79409, USA; grant.tinsley@ttu.edu

**Keywords:** whole-food nutrients, absorption, zinc, postbiotic

## Abstract

Biotransformation of minerals via glycosylation by microorganisms such as yeast and/or probiotics yields nutrients bound to a food matrix, resulting in increased bioavailability. The purpose of this study was to compare the effects of glycoprotein matrix-bound zinc (GPM) on absorption compared to inorganic zinc oxide. Sixteen participants ingested 11 mg of zinc as either GPM™ Soy-Free Zinc (GPM, Ashland, Kearny, NJ, USA) or zinc oxide (USP). Blood samples were taken at 0 (i.e., baseline), 30, 60, 90, 120, 180, 240, 300, 360, 420, and 480 min post-ingestion. GPM zinc concentrations were significantly higher at 120 min (*p* = 0.02; 12.4 ± 5.1 mcg/dL), 180 min (*p* = 0.002; 16.8 ± 5.1 mcg/dL), and 240 min (*p* = 0.007; 14.6 ± 5.1 mcg/dL) in comparison to USP zinc oxide. In addition, GPM zinc significantly increased iAUC by 40% (5840 ± 2684 vs. 4183 ± 1132 mcg/dL * 480 min, *p* = 0.02), and Cmax values were 10% higher in GPM compared to USP (148 ± 21 mcg/dL vs. 135 ± 17.5 mcg/dL, *p* = 0.08). Tmax was 12% slower in GPM compared to USP (112.5 ± 38.7 min vs. 127.5 ± 43.1 min); however, differences in Tmax failed to reach statistical significance (*p* = 0.28). Zinc bound to a glycoprotein matrix significantly increased absorption compared to zinc oxide.

## 1. Introduction

The essential trace mineral zinc has been linked to numerous health benefits, including maintaining healthy immune function and blood sugar levels, slowing macular degeneration, and promoting bone homeostasis and normal skeletal growth [1]. Zinc is a cofactor for more than 300 enzymes that rely on zinc to be able to catalyze chemical reactions [2]. In addition, zinc also contributes to the structure of important proteins and is involved in the regulation of gene expression [3,4]. Dietary intake or supplementation with zinc is necessary to maintain zinc homeostasis [5]. Zinc is absorbed in the distal small intestine [6] and the human body can maintain homeostasis over a broad exposure range. Corresponding zinc transporters on the apical and basolateral membrane of enterocytes are involved in the process and regulate cellular and body zinc homeostasis together with the cellular zinc-binding protein metallothionein [7,8]. Zinc is deposited in bone and skeletal muscle, where nearly 90% of zinc stores can be found; however, these tissues are not involved in the maintenance of homeostasis, as the turnover in these tissues is low [9].

Good dietary sources of zinc are red meat, some seafoods, dairy products, nuts, seeds, dried legumes, and whole-grain cereals [10,11,12]. Phytates, the storage form of phosphorus in plants, bind some minerals such as zinc in the intestine and form an insoluble complex, thereby limiting the absorption of zinc from foods [13]. One way to improve the absorption of zinc from foods is through reducing the amount of phytates through fermentation [14,15,16]. Fermentation of cassava with lactic acid bacteria has been shown to enhance zinc bioavailability in a preclinical model, where higher zinc levels were observed in the femur, serum, and liver of animals that consumed a lactic acid bacteria-fermented diet [17].

Nearly 2 billion people are at risk for zinc deficiency, mainly due to inadequate intake and absorption of zinc. Prolonged and severe zinc deficiency can result in severe negative effects on different aspects of health, including cognitive impairment, infertility, growth retardation, a depressed immune system, and symptoms of neurological dysfunctions like depression, confusion, forgetfulness, a lack of focus and mental clarity, and behavioral changes [18]. Zinc deficiency is common in developing countries due to malnutrition and is associated in developed countries with various illnesses such as Crohn’s disease and aging. It can occur from inadequate dietary intake (strict vegetarian diets or anorexia nervosa), excessive loss (diarrhea, diuretics, excessive alcohol consumption), increased metabolic demand (during pregnancy and lactation), and reduced ability to absorb zinc (certain medication such as antibiotics, co-ingestion with phytates or calcium) [13].

Glycoprotein matrix (GPM) nutrients are produced from a nutrient-dense broth, which is cultured and bio-transformed via glycosylation by microorganisms such as yeast and/or probiotics. The incorporation of minerals into a food matrix increases their absorption. The *lactobacilli* and *bifidobacteria* used for fermentation remain in the final product as postbiotics and the beta-glucan from yeast further enhance the benefits of zinc for immune health. In addition, glycoprotein matrix-bound nutrients are expected to have a slower and more sustained release compared to standard mineral salts.

The overall purpose of this study was to compare the effects of GPM-bound nutrients on the bioavailability of inorganic zinc oxide, commonly used as a dietary ingredient. The investigators hypothesized that fermentation would result in greater absorption and appearance in the blood following acute ingestion while potentially reducing the incidence of gastrointestinal distress.

## 2. Materials and Methods

A double-blind, randomized crossover study was conducted at the University of Mary Hardin-Baylor (UMHB), Belton, TX, USA, in accordance with the Declaration of Helsinki guidelines. All procedures were approved by the Institutional Review Board of the University of Mary Hardin-Baylor (IRB approval number 267, date of approval: 4 October 2023). This study was retrospectively registered with the ISRCTN registry on 2 February 2024 as ISRCTN67008850.

### 2.1. Participants

Males (n = 8) and females (n = 8) between the ages of 18 and 55 years were recruited to participate in this study. In order to qualify for this study, participants needed to have normal body weight [body mass index (BMI) of 19–24.99 kg/m^2^] and be recreationally active (according to American College of Sports Medicine Guidelines). Participants were not allowed to consume any nutritional supplements known to affect measures of the current study for 6 weeks prior to the study, including probiotics, prebiotics, and digestive enzymes. Exclusion criteria included any individual who was concurrently being treated for or had been diagnosed with a gastrointestinal, cardiac, respiratory, circulatory, musculoskeletal, metabolic, immune, autoimmune, psychiatric, hematological, neurological, or endocrinological disorder. Participants who were determined to not be weight-stable (defined as measured body mass deviating by 2% or more in the previous 30 days) and participants who were not willing to abstain from alcohol, nicotine, and caffeine for 12 h prior to each visit were excluded. Participants were randomized using Random.org to first consume one of the two conditions, U.S. Pharmacopeia-grade zinc oxide (USP) or glycoprotein matrix-bound zinc (GPM), followed by the other condition after the wash-out period.

### 2.2. Experimental Protocol

On the day of experimental testing, participants arrived at the laboratory following an overnight fast. All experimental testing sessions occurred between 7 a.m. and 8 a.m., and individual participant start times were repeated on the second arm of the crossover design. The occurrence of adverse events was recorded throughout completion of the study visits. Adverse events were collected through spontaneous reporting by the study participants, through clinical evaluation or interaction by a research team member with a study participant, and through questionnaires prior to and 480 min post-ingestion. The GI health questionnaires evaluated stomach ache, abdominal pain or cramps, bloating, subjective impression of rectal gas excretion, and nausea, and ranked side effects on a scale from 0 (no symptoms) to 5 (severe symptoms). In addition, participants were asked to rank the severity of dizziness, headache, fast or racing heart rate, heart skipping or palpitations, shortness of breath, nervousness, blurred vision, and other unusual or adverse effects on a scale from 0 (none) to 5 (very severe). Participants rested semi-supine for placement of a Teflon catheter into an antecubital vein for multiple blood samplings. The catheter was kept patent by flushing with 2–3 mL of 0.9% sodium chloride. Following baseline sampling, participants ingested their respective supplement with 177 mL of cold water. Thereafter, blood samples were taken at 30, 60, 90, 120, 180, 240, 300, 360, 420, and 480 min post-ingestion. Whole blood was collected and transferred into Becton Dickson (BD) 6.0 mL tubes (BD EDTA Vacutainer Trace Metal-Free) for obtaining plasma and was subsequently centrifuged at 1500× *g* for 15 min at 4 °C. Resulting plasma was then aliquoted into Quest Diagnostic “Trace Element and Metal-Free Vial” and transported at room temperature to Quest Diagnostic for analysis. Participants ingested the supplement with 177 mL of water immediately after the initial blood collection. The participant then received 59 mL of water every 60 min until hour 4, when the participant received a zinc-free snack, and then again at every hour for the remaining 4 h. The snack included two packages of Cheez-it™ Snack Mix (21 g each) and one Ocean Spray™ Apple Juice (118 mL). The total caloric intake was 250 calories, with 4 g of protein, 42 g of carbohydrates, and 7 g of fat. Subsequently, a 1-week wash-out period was implemented before participants were crossed over to the other supplement and repeated the experimental procedure.

### 2.3. Plasma Zinc Analysis

Plasma samples were diluted with a solution of nitric acid and an internal standard, and then were analyzed for plasma zinc levels using Inductively Coupled Plasma/Mass Spectrometry (ICP/MS) (Quest Diagnostics Nichols Institute, Valencia, CA, USA). A multi-point calibration was performed with each assay run, and the assay has a clinical reporting range of 100–10,000 mcg/L. All reagents utilized were Optima grade or higher, and calibrators were NIST-traceable and manufactured in an ISO 17034/17025 facility specifically designed for Quest Diagnostics [19,20]. An extensive interference study was performed during the validation of the assay and no interfering substances were identified. All other assay specifics and settings for these analyses are proprietary in nature.

### 2.4. Supplementation

Both zinc treatments, GPM (220 mg GPM™ Soy-Free Zinc containing 5% zinc, Ashland, Kearny, NJ, USA) and USP (zinc oxide), contained the equivalent of 11 mg of zinc, 100% of the daily value for adults based on the FDA guidelines (Reference Guide: Daily Values for Nutrients), and were administered in the form of one uncoated tablet. The glycoprotein matrix was produced from a carbohydrate and protein source using *Saccharomyces cerevisiae* yeast strains and heat during the initial culturing phase. After fermentation for several hours, the active nutrient (zinc oxide) was added to the cultured broth, initiating the growth phase. The yeast absorbed the zinc and incubated it aerobically into its cell structure, creating a matrix around the active nutrient. During the final autolysis phase, enzymes and probiotic bacteria were added at low heat for several hours to finish the digestion process. After several hours, the product was further processed to yield the final powdered glycoprotein-bound active nutrient.

### 2.5. Statistical Analysis

Initially, zinc concentrations were examined for extreme outliers (i.e., values above Q3 + 3 × IQR or below Q1 − 3 × IQR) and no extreme outliers were present. Data were subsequently analyzed in R (version 4.3.1) using linear mixed-effects models (*nlme* package, v. 3.1-162) with a random intercept for participant and a first-order autoregressive (AR1) variance–covariance matrix [21]. These models were fit by maximizing the restricted log-likelihood (REML). Model assumptions were examined through graphical methods (i.e., residuals vs. fitted plots and quantile–quantile plots). Fixed effects of condition and time, along with their interaction, were examined. As needed, significant effects were followed up with pairwise comparisons using the *emmeans* package (v. 1.8.7), applying the false discovery rate correction for multiple comparisons [22]. Data were visualized using the *ggplot2* (1) package (v. 3.4.2) with within-subject error bars for line plots [23,24].

For the pharmacokinetic analysis, the incremental area under the concentration vs. time curve (iAUC) was calculated using the method of Brouns et al. [25]. The *PKNCA* package (v. 0.10.2) was used to establish the maximum observed concentration (Cmax) and time of maximum observed concentration (Tmax) [26]. No extreme outliers were present when iAUC and Cmax values were evaluated. The iAUC and Cmax values were analyzed with paired-samples t-tests and Cohen’s *d* effect sizes, using the *rstatix* package (v. 0.7.2) [27]. The normality of models was examined using graphical methods. Due to the nature of the data, Tmax values were analyzed using the non-parametric Wilcoxon signed-rank test. Significant effects of condition were followed up using pairwise t-tests. For all tests, statistical significance was accepted at *p* < 0.05, and *p* values between 0.05 and 0.1 were considered trends. Side effects were not analyzed inferentially due to 96.7% of data points having values of zero.

## 3. Results

### 3.1. Participants

**Figure 1:**
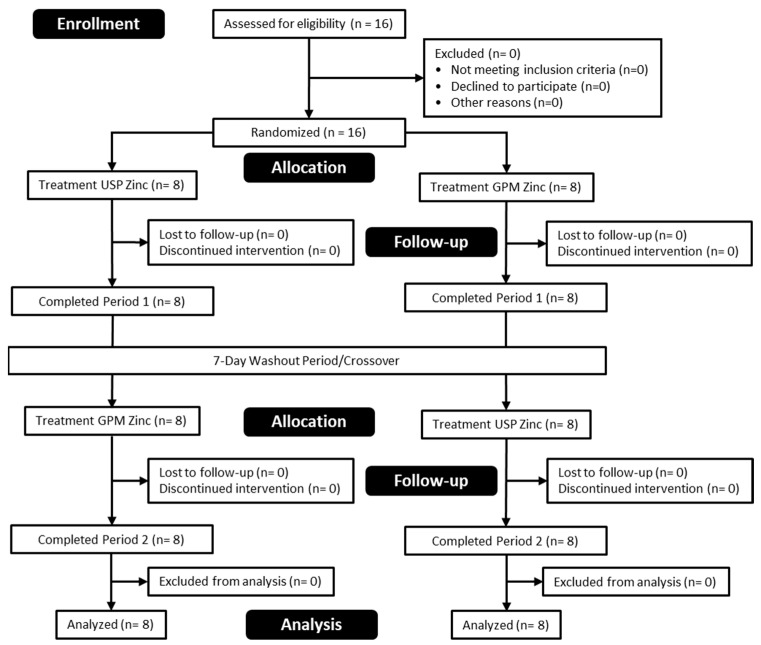
CONSORT flow diagram.

A total of 16 healthy men and women were enrolled and successfully completed the study protocol. Participant characteristics are shown in Table 1 and a CONSORT diagram is provided in Figure 1.

### 3.2. Plasma Zinc Concentrations

**Figure 2:**
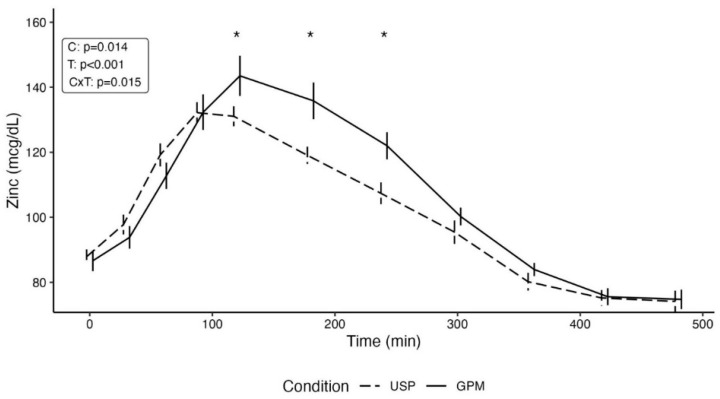
Zinc concentrations over the duration of the experimental testing sessions are displayed. Following the significant condition by time interaction, pairwise comparisons were performed to evaluate potential differences between conditions at distinct timepoints. Asterisks indicate a statistically significant (*p* < 0.05) difference between conditions at the specified timepoint. Abbreviations: C—condition main effect; CxT—condition by time interaction; GPM—glycoprotein matrix-bound zinc; T—time main effect; USP—USP zinc oxide.

For raw zinc concentrations, a statistically significant condition by time interaction was observed (*p* = 0.02). Pairwise comparisons indicated that there were no differences between conditions at baseline (*p* = 0.84), 30 min (*p* = 0.48), 60 min (*p* = 0.24), or 90 min (*p* = 0.98) following supplement ingestion. However, statistically significant differences between conditions were observed at 120 min (*p* = 0.02; 12.4 ± 5.1 mcg/dL [mean ± SE]), 180 min (*p* = 0.002; 16.8 ± 5.1 mcg/dL), and 240 min (*p* = 0.007; 14.6 ± 5.1 mcg/dL), with higher values in the GPM condition (Figure 2). Subsequently, no differences between conditions were observed at 300 min (*p* = 0.39), 360 min (*p* = 0.50), 420 min (*p* = 0.95), or 480 min (*p* = 0.92) following supplement ingestion.

### 3.3. Pharmacokinetics

**Figure 3:**
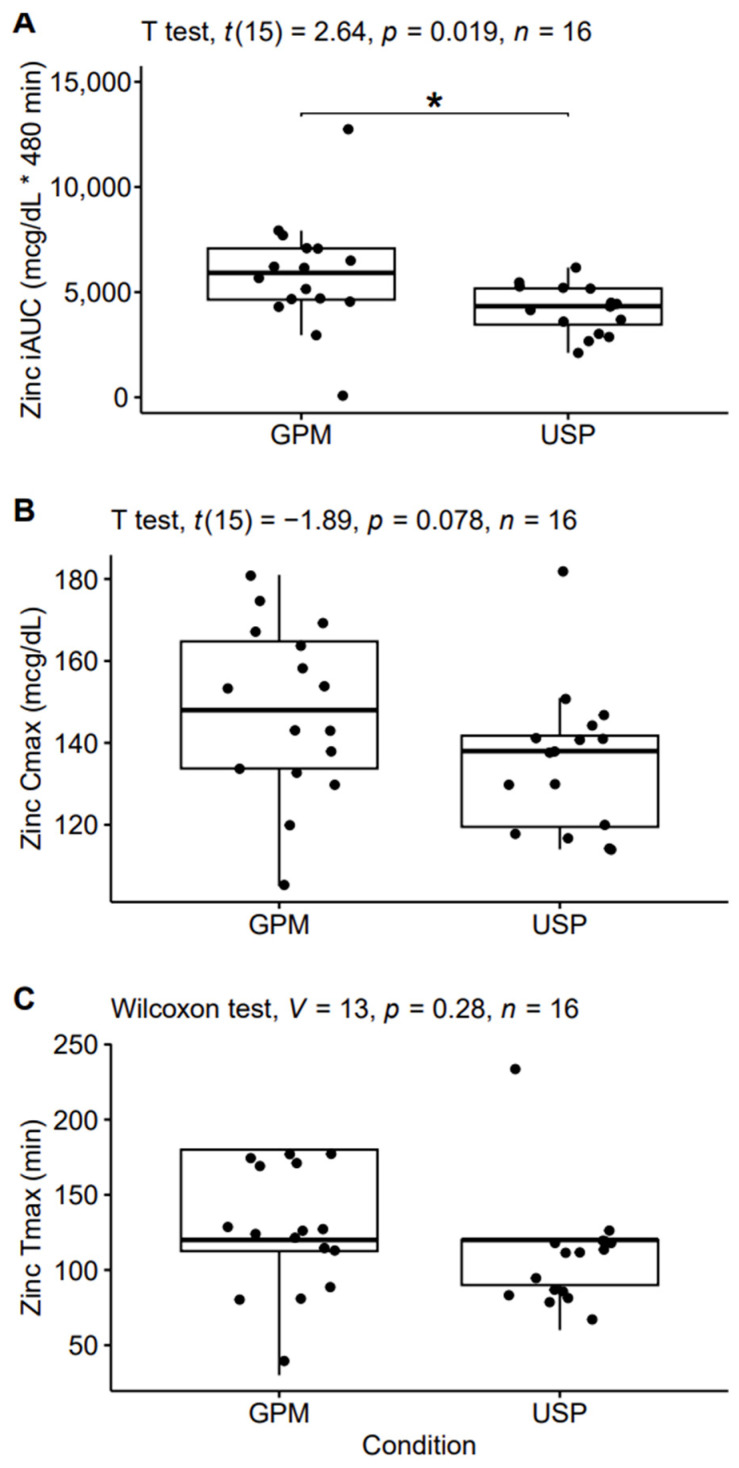
Pharmacokinetic analysis. The incremental area under the curve (iAUC; panel **A**), maximum observed concentration (Cmax; panel **B**), and time of Cmax (Tmax; panel **C**) are displayed. Asterisks indicate statistically significant (*p* < 0.05) differences between conditions. Abbreviations: GPM—glycoprotein matrix bound zinc; USP—USP zinc oxide.

In the pharmacokinetic analysis, a significant difference between conditions was observed for iAUC (*p* = 0.02; Cohen’s *d* = 0.66 [moderate]), with 40% larger values in the GPM condition, on average (5840 ± 2684 vs. 4183 ± 1132 mcg/dL X 480 min [mean ± SD]; Figure 3). A trend for a difference between conditions was observed for Cmax (*p* = 0.08; Cohen’s *d* = 0.47 [small]). On average, Cmax values were 10% higher in GPM compared to USP (148 ± 21 mcg/dL vs. 135 ± 17.5 mcg/dL). Tmax was 12% slower in GPM compared to USP (112.5 ± 38.7 min vs. 127.5 ± 43.1 min); however, differences in Tmax failed to reach statistical significance (*p* = 0.28).

### 3.4. Adverse Event Monitoring

Both supplements were generally well-tolerated and participants reported minimal side effects.

#### 3.4.1. GPM Zinc

GI health questionnaires revealed no stomach ache, cramps, bloating, subjective impression of rectal gas excretion, or nausea. One participant in each group reported abdominal pain at baseline but not at the 480 min timepoint. No incidents of dizziness, fast or racing heart rate, heart skipping or palpitations, shortness of breath, nervousness, blurred vision, or other unusual or adverse effects were reported at either timepoint (0 and 480 min). No incidences of headache were reported at baseline. At the 480-min timepoint, 50% of the participants reported a headache with a severity of 1 to 3 out of 5 in the GPM group.

#### 3.4.2. USP Zinc

GI health questionnaires revealed no stomach ache, cramps, bloating, subjective impression of rectal gas excretion, or nausea. One participant in each group reported abdominal pain at baseline but not at the 480-min timepoint. In addition, one participant reported nervousness and blurred vision, rated at 1 out of 5 each, and one participant reported dizziness, rated at 2 out of 5, at the 480 min timepoint in the USP group. No incidences of headache were reported at baseline and 38% of the participants reported a headache with a severity ranging from 1 to 4 out of 5 in the USP group at the 480 min timepoint.

## 4. Discussion

In the present study, double fermentation with yeast and probiotic strains significantly increased the absorption of zinc compared to zinc oxide, as indicated by the 40% greater iAUC and 10% greater Cmax, as well as the significantly higher zinc concentrations from 2 to 4 h following ingestion.

Zinc absorption depends on the amount of circulating zinc and zinc content in the intestine. Zinc-deficient individuals absorb more zinc compared to a zinc-sufficient subject. While zinc absorption from food is negatively correlated with phytate and fiber content, organic and inorganic salts of zinc (e.g., acetate, citrate, phosphate, sulfate, or chloride), or complexes with amino acids (e.g., histidine), can increase absorption to varying degrees. Acute absorption of 15 mg elemental zinc complexed with histidine at a ratio of 1:2 was shown to be at least equivalent to a 45 mg dose of zinc sulfate [28]. Acute oral absorption of 50 mg elemental zinc in the form of zinc oxide showed lower absorption compared to zinc acetate based on solubility–dissolution factors [29]. A comparison of the oral bioavailability between 15 mg elemental zinc in the form of bis-glycinate salt and gluconate salt after 8 h resulted in bis-glycinate significantly increasing the oral bioavailability of zinc compared with the gluconate form [30]. Another acute study compared the absorption of 10 mg elemental zinc in the form of oxide, gluconate, and citrate salt using a double-isotope tracer method with ^67^Zn and ^70^Zn to measure zinc absorption, showed lower zinc absorption from zinc oxide compared to zinc gluconate and zinc citrate [31].

In addition to improving the absorption of zinc through the concept of increasing the solubility by creating different zinc salts, another successful method to enhance the micronutrient bioavailability is through fermentation of plant-based foods [32]. A single dose of 20 mg elemental zinc-enriched yeast and gluconate supplements were evaluated over a 48 h period, showing that the gluconate salt resulted in higher zinc concentrations in the blood during the first 6 h but also revealed greater losses in the feces, whereas the yeast form also increased concentrations in the blood with time but showed significantly less loss in the feces [33].

Fermented foods can also comprise probiotic bacteria, specifically the lactic acid-producing type, which enhance nutritional attributes by improving mineral bioavailability and forming bioactive compounds [34]. The consumption of fermented foods is frequently connected with several health benefits. Plant-derived food micronutrient bioavailability is usually improved by fermentation, which provides better shelf-life to foods with enhanced gut microbial ecology that promotes a healthy status. *Lactobacillus* and *Lactococcus* are the most frequently used lactic acid bacteria cultures, although others, such as yeast, are also used for fermentation purposes [35]. The regular consumption of fermented foods has been described to improve the immune system, reducing the probability of developing morbidities due to constant communication between bacteria and host immune system [36]. This communication changes the microbial composition of the intestine, maintaining controlled pathogenic microflora while also supporting beneficial microbe populations [37].

The GPM zinc analyzed in this study was produced using *lactobacillus* and *bifidobacteria* strains. Due to the heat during the drying process, the probiotics used during manufacturing were not viable (heat-killed); however, they remained in the final product. Probiotics are living cells that have the ability to grow, divide, and reproduce. Keeping probiotics alive in commercial products is a major challenge, as probiotics are sensitive to water, heat, and other ingredients that are commonly found in nutritional products such as zinc. Postbiotics (derived from Greek, ‘post’, meaning after, and ‘bios’, meaning life) are dead bacteria or cell fragments, generated through a deliberate “killing” process such as lysis, pressure, radiation, or, in the case of GPM, heat. Isolated metabolites that are produced by microorganisms, such as butyrate, no longer meet the criteria for a postbiotic [38]. Postbiotics, inanimate microorganisms, and microbial cell fragments have been shown to provide numerous health benefits, including improved nutrient absorption and immune and gastrointestinal health, even while the bacteria are no longer alive [39,40].

In addition to postbiotics, GPM contains beta-glucan from yeast, which has been widely used to promote a healthy immune response. Beta-glucans are naturally occurring polysaccharides that are produced by bacteria, yeast, fungi, and many plants. Chemically, beta-glucan is a non-starch polysaccharide with repeating units of glucose. The glucose units may branch in several ways, depending upon the source from which beta-glucan is extracted. These glucose polymers may consist of a backbone of beta-(1-3)-linked beta-D-glucopyranosyl units with beta-(1-4)- or beta-(1-6)-linked side chains of varying distribution and length. Beta-glucans from bacteria are linear beta-(1-3)-glucans without any branching (*Agrobacterium* sp. *R259*, *Alcaligenes faecalis*, *Euglena gracilis*), while fungi are β-(1-3)-glucans with short β-1,6 branching (*Pleurotus ostreatus*, *Schizophyllum commune*, *Sclerotium glucanicum*, *Lentinus edodes*), yeast (*Saccharomyces cerevisiae*) are β-(1-3)-glucans with long β-1,6 branching, and beta-glucans from oat or barley are linear with β-(1-4) linkage with shorter stretches of β-(1-3) linkage (and are soluble). Numerous factors such as the molecular structure, size, conformation, and solubility influence the activity of beta-glucan [41]. Supplementation with yeast-derived beta-glucan has been shown to improve immune health following strenuous exercise [42] and may protect against upper respiratory tract infections (URTI) and reduce the duration of URTI symptoms in elderly people [43]. Similar to postbiotics, beta-glucan can positively influence the gut microbiota and nutrient absorption [44].

The current study investigated whether zinc in a food-style format as glycoprotein would reduce the gastrointestinal discomfort previously reported for inorganic zinc salts. Both forms of zinc were very well-tolerated, and no gastrointestinal discomfort was reported in either group. Fasting headache, non-pulsating pain of mild to moderate intensity, is common during fasting and is resolved after food consumption [45]. Caffeine withdrawal and hypoglycemia have been implicated as causative factors [46]. Habitual consumers of caffeine experience moderate to severe headaches, nausea, and cognitive deficits due to rebound dilatation of cerebral vasculature caused by increased sensitivity to the potent cerebral vasodilator adenosine [47]. Participants in this study reported to the lab following an overnight fast, with 14 out of 16 indicating they were regular caffeine users with daily consumption exceeding 100 mg. Headaches have been reported with high-dose zinc intake; however, the tolerable upper intake levels, the largest amount of zinc that a person can take each day with little to no associated risk, was established at 40 mg per day for adults, which is well above the amounts ingested in this study [48].

The strengths of the current study included the crossover design, accounting for intersubject variability of nutrient absorption, and the amount of elemental zinc (11 mg) equaling 100% of the daily value, whereas most previous studies with zinc salts and amino acid complexes used higher doses (e.g., 15 mg) that exceeded the recommended daily intake. Limitations include the lack of collection of fecal material to analyze zinc retention. Endogenous zinc is excreted into the intestinal lumen, from where it is reabsorbed, or excreted, with feces. The quantity of fecal zinc is determined by both the quantity of recently absorbed zinc and zinc status. Future studies should include comparisons to more easily absorbed forms of zinc such as zinc bisglycinate, citrate, or acetate.

## 5. Conclusions

Zinc bound to a glycoprotein matrix significantly increases absorption compared to zinc oxide when observed as peak concentration and area under the curve. This observed improvement in absorption was well-tolerated in the gastrointestinal tract.

## Figures and Tables

**Table 1:** Participant characteristics at baseline (n = 16). Data are presented as means ± standard deviation.

	Mean Age (years)	Mean Height (cm)	Mean Weight (kg)
Male (n = 8)	22.5 ± 3.8	182.6 ± 5.4	89.1 ± 4.0
Female (n = 8)	26.8 ± 6.9	166.7 ± 4.7	76.0 ± 8.5
Total (n = 16)	24.6 ± 5.9	174.6 ± 9.5	82.5 ± 9.3

## Data Availability

The research data are available from the corresponding author upon request.
